# Millions of excess cases and thousands of excess deaths of malaria occurred globally in 2020 during the COVID-19 pandemic

**DOI:** 10.7189/jogh.12.05045

**Published:** 2022-12-17

**Authors:** Qiao Liu, Wenxin Yan, Chenyuan Qin, Min Du, Min Liu, Jue Liu

**Affiliations:** 1 Department of Epidemiology and Biostatistics, School of Public Health, Peking University, Beijing, China; 2 Global Center for Infectious Disease and Policy Research, Global Health and Infectious Diseases Group, Peking University, Beijing, China; 3 Institute for Global Health and Development, Peking University, Beijing, China

## Abstract

**Background:**

The COVID-19 pandemic disrupted malaria-related health care services, leading to an excess burden of malaria. However, there is a lack of research on the indirect global impact of the COVID-19 pandemic on malaria. We aimed to assess the excess burden of malaria due to the COVID-19 pandemic in malaria-endemic countries in 2020.

**Methods:**

Based on data from the World Health Organization Global Observatory, we used estimated annual percentage changes (EAPCs) from 2000 to 2019 (model A) and from 2015 to 2019 (model B) to predict the malaria burden in 2020. We calculated the ratios between reported and predicted malaria incidence (incidence rate ratio (IRR)) and mortality rates (mortality rate ratio (MRR)).

**Results:**

In 2020, African countries suffered the most from malaria, with the largest number of malaria cases (64.7 million) and deaths (151 thousand) observed in Nigeria. Most countries showed a decrease in malaria incidence and mortality rates from 2000 to 2019, with the strongest decline in incidence rates in Bhutan (EAPC = -35.7%, 95% CI = -38.7 to -32.5%) and mortality rates Ecuador (EAPC = -40.6%, 95% confidence interval (CI) = -46.6 to -33.8%). During the COVID-19 pandemic in 2020, there was a total of 18 million excess malaria cases and 83 291 excess deaths per model A, and 7.4 million excess cases and 33 528 excess deaths per model B globally. Malaria incidence rates increased excessively in over 50% of the malaria-endemic countries, with the greatest increase in Costa Rica (IRR = 35.6) per model A and Bhutan (IRR = 15.6) per model B. Mortality rates had increased excessively in around 70% of the malaria-endemic countries, with the greatest increase in Ecuador in both model A (MRR = 580) and model B (MRR = 58).

**Conclusions:**

The emergence of the COVID-19 pandemic indirectly caused an increase in the prevalence of malaria and thwarted progress in malaria control. Global efforts to control the pandemic’s impact should be balanced with malaria control to ensure that the goal for global malaria elimination is achieved on time.

Despite being preventable and curable, malaria is still considered a major public health problem and significantly impacts people’s health and livelihoods globally, especially in malaria-endemic countries. The World Health Organization (WHO) estimated that 241 million malaria episodes occurred worldwide in 2020, which is 14 million more than in 2019, mostly in the WHO African Region (95%) [1]. WHO also reported an estimated 627 000 malaria deaths in 2020, a 12% increase from 2019, with children under the age of five accounting for 77% of all malaria deaths across the world [1]. The increase in malaria episodes and deaths in 2020 compared with 2019 caused global concern. The Global Technical Strategy for Malaria 2016-2030 (GTS), proposed by WHO in 2015, set its first milestone in 2020 to reduce malaria incidence and mortality rates by at least 40% compared with 2015; however, the progress had stalled or trends reverted for global malaria incidence and mortality [2,3]. A previous study demonstrated increasing trends in malaria age-standardized incidence rate (ASIR) in countries with high-middle, middle, and low-middle Socio-demographic Indices, and the uptrends remained in 2019 [4]. This study also raised concerns that 40 countries had a higher ASIR in 2019 than in 2015 [4].

As first reported in 2019, coronavirus disease 2019 (COVID-19), caused by severe acute respiratory syndrome coronavirus 2 (SARS-CoV-2) and its various emerging variants, is posing a very high health threat in sustained pandemic waves. To date, 635 million confirmed cases and 6.61 million deaths caused by COVID-19 were reported worldwide [5]. The COVID-19 pandemic, declared a global health emergency by the WHO, created an enormous strain on health systems worldwide [6,7]. There is increasing evidence showing that the COVID-19 pandemic adversely impacted on the provision of a wide range of essential health services, especially in low- and middle-income countries [8-10]. A previous study observed significant disruption to the prevention, diagnosis, treatment, and management of tuberculosis, HIV, and dengue fever due to the COVID-19 pandemic in certain countries [6].

Meanwhile, previous studies have shown that COVID-19 also adversely impacted health services for malaria. Malaria diagnoses decreased by 56% and malaria treatment services plummeted by 59% in Bangladesh, Cambodia, India, Indonesia, Lao, Pakistan and the Philippines, for April to September 2020, compared with the same period in 2019 [11]. Consequently, the malaria burden in 2020 was worrisome. A modelling study showed that, if malaria prevention activities were halted, the malaria burden in 2020 could be more than double that of 2019 [12]. Compared to the same period in 2017, 2018, and 2019, there was an excess of over 30 000 malaria cases from January to June 2020 in Zimbabwe [13]. Moreover, another modelling analysis found that under pessimistic scenarios, malaria mortality in Africa could be almost doubled due to COVID-19-related disruption to malaria control [14]. An estimated 68% of the additional malaria deaths in 2020 compared with 2019 were due to health service disruptions during the COVID-19 pandemic [1].

The COVID-19 pandemic disrupted malaria-related health care services, leading to an excess burden of malaria. However, there is a lack of research on the indirect global impact of the COVID-19 pandemic on malaria. With the health service disruptions, government policy responses, and all other societal issues caused by the COVID-19 pandemic, global progress in halting malaria cases and deaths could be derailed. We aimed to assess the excess incidence and mortality of malaria in malaria-endemic countries in 2020 during the COVID-19 pandemic, using malaria data from the WHO Global Health Observatory data set. Our findings can serve to extend and complement previous studies and help improve the understanding of the pandemic’s indirect impacts on malaria. We also expect to draw attention to the need for sustained efforts to control malaria amidst the pandemic and meet the global goal of malaria elimination.

## METHODS

### Data collection

We obtained malaria incidence and mortality data from the WHO Global Health Observatory Data set [15] and extracted annual malaria incidence and mortality data by countries with their 95% uncertainty intervals (UIs) from 2000 to 2020.

The general methodological approaches for estimating the malaria burden are described elsewhere [1]. Briefly, we used different methods for estimating the burden in each country with different malaria transmission statuses. Three different methods were used for estimating malaria cases, divided by the following conditions: 1) countries and areas outside the WHO African Region and low transmission countries and areas in the African Region, 2) high transmission countries in the WHO African Region and countries in the WHO Eastern Mediterranean Region in which the quality of surveillance data did not permit a robust estimate for the number of reported cases, and 3) elimination countries and countries at the stage of prevention of reintroduction. We also used three different methods for estimating malaria deaths in the following conditions: 1) low transmission countries and areas, both within and outside Africa, 2) countries in the WHO African Region with a high proportion of deaths due to malaria, and 3) the number of indigenous malaria deaths registered by national malaria programs is reported without further adjustments [1].

### Statistical analyses

Estimated annual percentage change (EAPC) is widely used to quantify the rate trend over a specific interval. We fitted a regression line to the natural logarithm of the rates (*y = α + βx + ε*, where *y* = ln (rate) and *x* = calendar year). EAPC was calculated as 100 × (*e^β^* − 1), with 95% confidence intervals (CIs) obtained from the linear regression model. We calculated overall EAPC by the annual incidence and mortality rates of malaria in malaria-endemic countries. We used two models to calculate the EAPC: model A used malaria incidence and mortality rates from 2000 to 2019, while model B used malaria incidence and mortality rates from 2015 to 2019.

We separately fitted the calendar year 2020 into the regression lines of model A and B and predicted the malaria incidence and mortality rates in 2020. We calculated incidence rate ratios (IRRs) and mortality rate ratios (MRRs) by the formula: *Incidence (mortality) rates in 2020 reported by WHO/Predicted incidence /mortality) rates in 2020*

We estimated the predicted malaria cases and deaths in 2020 by the formula: *cases or deaths in 2020 reported by WHO/IRR or MRR*. We then compared the predicted malaria data with actual malaria data reported by the WHO to see the indirect impacts of the COVID-19 pandemic on malaria.

We performed all the statistical analyses using the R program (version 4.4.1).

## RESULTS

### Global malaria burden in 2019 and 2020

According to the WHO World Malaria Report 2020, there were 84 malaria-endemic countries in 2020 globally. After excluding three countries where the malaria incidence rates were zero in 2019 (Belize, Cabo Verde, and Timor-Leste), a total of 81 malaria-endemic countries were included in our analysis.

**Figure 1:**
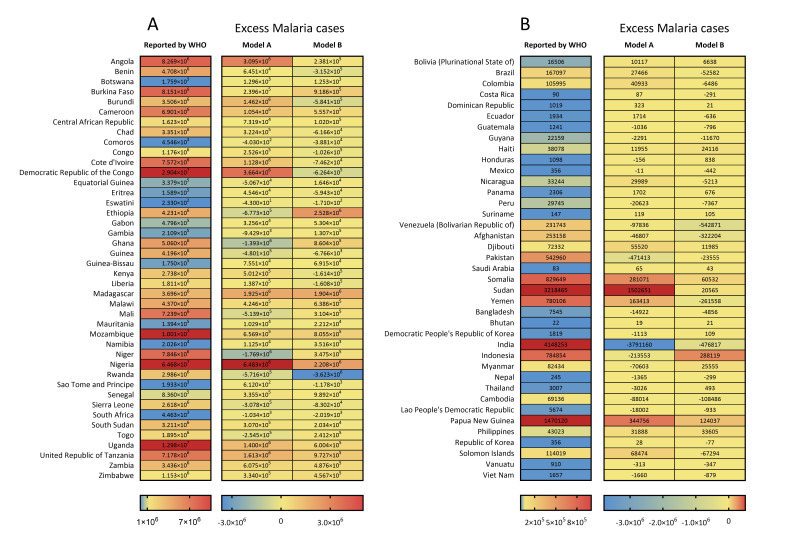
Heatmaps of reported and excess malaria cases (by model A and model B) in 2020 in malaria-endemic countries. **Panel A:** African countries. **Panel B:** countries outside Africa. WHO – World Health Organization.

**Figure 2:**
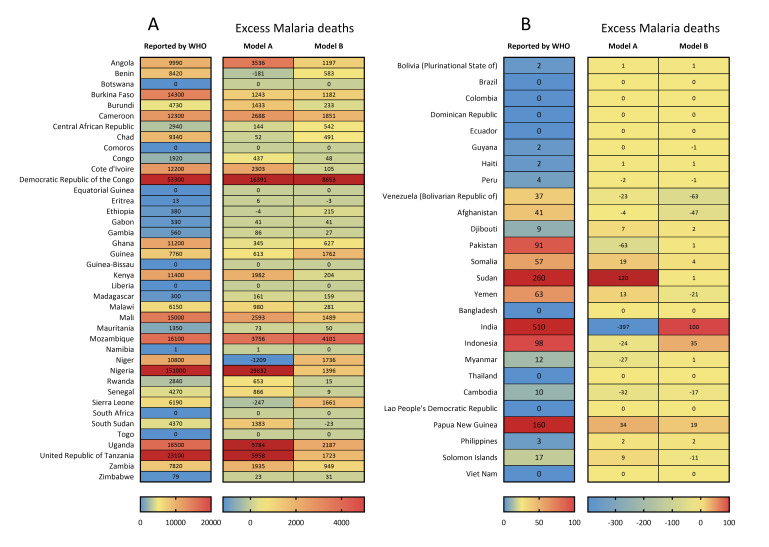
Heatmaps of reported and excess malaria deaths (by model A and model B) in 2020 in malaria-endemic countries. **Panel A:** African countries. **Panel B:** countries outside Africa. WHO – World Health Organization.

**Figure 3:**
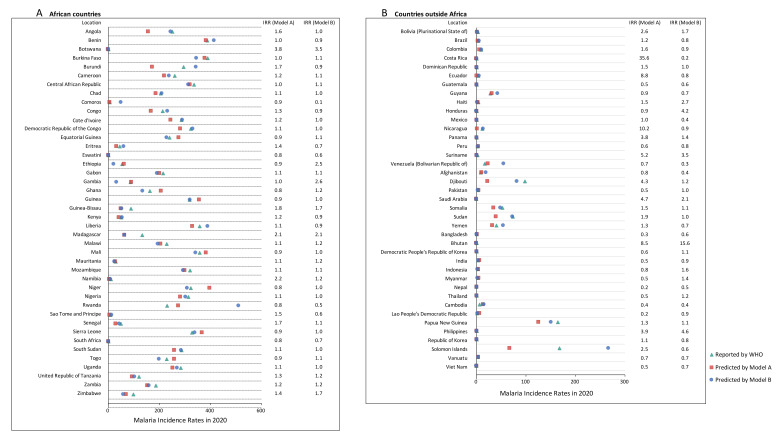
Reported and predicted malaria incidence rates (by models A and B) in 2020, and IRRs in malaria-endemic countries. **Panel A:** African countries. **Panel B:** countries outside Africa. IRR – incidence rate ratio, WHO – World Health Organization.

**Figure 4:**
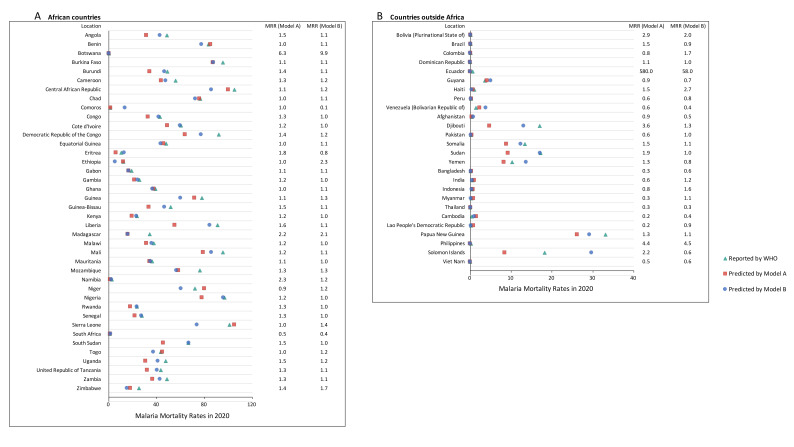
Reported and predicted malaria mortality rates (by models A and B) in 2020, and MRRs in malaria-endemic countries. **Panel A:** African countries; **Panel B:** countries outside Africa. MRR – mortality rate ratio, WHO – World Health Organization.

In 2020, the WHO African region suffered the most from malaria, with the highest number of malaria cases observed in Nigeria (64.7 million), followed by the Democratic Republic of the Congo (DR Congo) (29 million) and Uganda (13 million). (**Figure 1**, Panel A) In 2020, malaria caused the most deaths in Nigeria (151.0 thousand), followed by DR Congo (53.3 thousand) and Tanzania (23.1 thousand). (**Figure 2**, panel A) The highest malaria incidence rate in 2020 was 389.9 (95% UI = 241.2 to 593.8) per 1000 in Burkina Faso, followed by Benin (388.3 per 1000, 95% UI = 274.3 to 535.1) and Liberia (358.0 per 1000, 95% UI = 211.7 to 565.7). (**Figure 3**, panel A and Figure S1 in the **Online Supplementary Document**) Although the malaria incidence rates in the Central African Republic were relatively low, it had the highest mortality rates in 2020 (105.2 per 100 000, 95% UI = 61.0 to 193.8), followed by Sierra Leone (101.0 per 100 000, 95% UI = 77.6 to 128.6) and Nigeria (96.9 per 100 000, 95% UI = 73.4 to 133.3). (**Figure 4**, Panel A and Figure S1 in the **Online Supplementary Document**)

Several countries outside of the WHO African region could not be neglected due to the still worrisome malaria burden. India had the largest number of malaria cases at 4.1 million, followed by Sudan with 3.2 million and Papua New Guinea with 1.5 million (**Figure 1**, panel B). These countries also had the largest number of deaths due to malaria: 510 in India, 260 in Sudan, and 160 in Papua New Guinea. (**Figure 2**, panel B) In 2020, the highest incidence rates were reported in the Solomon Islands (167.7 per 1000, 95% UI = 146.0 to 198.0), followed by Papua New Guinea (164.3 per 1000, 95% UI = 112.7 to 221.1). (**Figure 3**, panel B and Figure S1 in the **Online Supplementary Document**) The Solomon Islands and Papua New Guinea, both located in the WHO Western Pacific region, also had the highest mortality rates of malaria in 2020 – 33.1 per 100 000 (95% UI = 1.8 to 64.3) in Papua New Guinea and 18.2 per 100 000 (95% UI = 2.5 to 29.8) in the Solomon Islands. (**Figure 4**, panel B and Figure S1 in the **Online Supplementary Document**) Meanwhile, Eastern Mediterranean countries such as Djibouti, Somalia, and Sudan also needed more attention, with malaria incidence rates over 50.0 per 1000 and mortality rates over 10per 100 000 in 2020.

### Trends of malaria incidence and mortality rates from 2000 to 2019 and from 2015 to 2019

**Figure 5:**
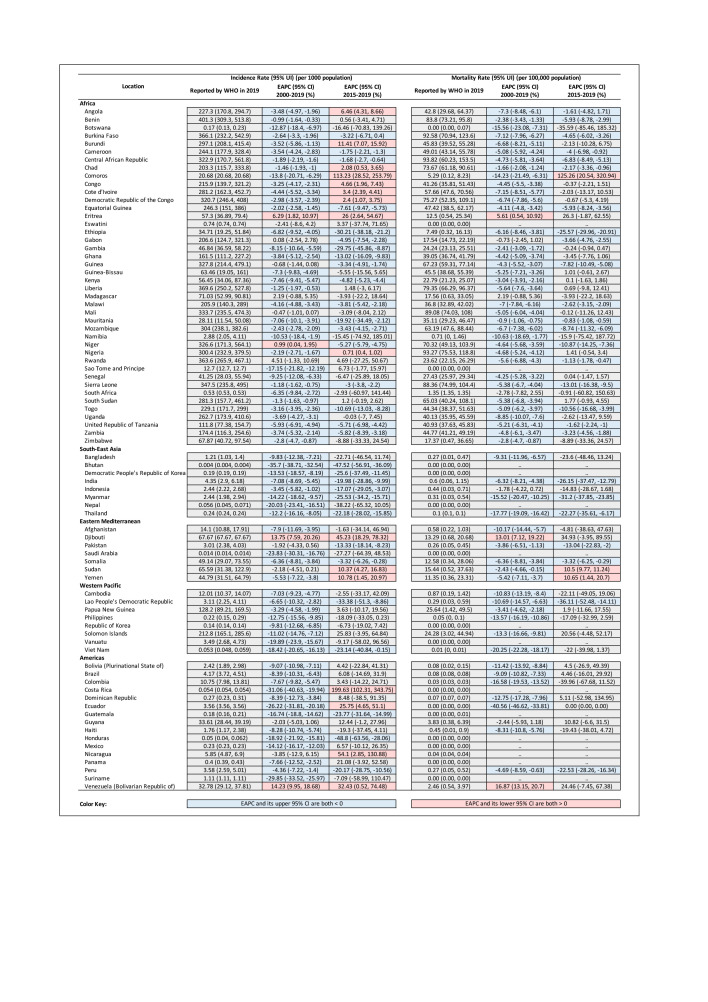
Malaria incidence and mortality rates in 2019 and EAPC (from 2000 to 2019, and from 2015 to 2019) in malaria-endemic countries. EAPC, estimated annual percentage change. CI – confidence interval, UI – uncertainty interval, WHO – World Health Organization.

As shown in **Figure 5**, most countries had downtrends of malaria incidence and mortality rates from 2000 to 2019, with the strongest decline in incidence rate occurring in Bhutan (EAPC = -35.7%, 95% CI = -38.7 to -32.5%) and the strongest decline in mortality rates in Ecuador (EAPC = -40.6%, 95% CI = -46.6 to -33.8%). However, in Venezuela, the malaria incidence rate increased by an average of 14.2% (95% CI = 9.9 to 18.7%) annually and the malaria mortality rate increased by an average of 16.9% (95% CI = 13.1 to 20.7%) per year from 2000 to 2019. Incidence and mortality rates in Djibouti and Eritrea also increased between 2000 and 2019; EAPCs of incidence rates were 13.7% (95% CI = 7.6 to 20.3%) in Djibouti and 6.3% (95% CI = 1.8 to 11.0%) in Eritrea and EAPCs of mortality rate were 13.0% (95% CI = 7.1 to 19.2%) in Djibouti and 5.6% (95% CI = 0.5 to 10.9%) in Eritrea.

From 2015 to 2019, the strongest decline in malaria incidence rates was observed in Honduras by an average of 48.8% (95% CI = 28.1 to 63.6%) per year and the strongest increase was in Costa Rica by an average of 199.6% (95% CI = 102.3 to 343.8%) per year. Malaria mortality rates declined the strongest in Laos by an average of 36.1% (95% CI = 14.1 to 52.5%) per year and the strongest increase in Comoros by an average of 125.3% (95% CI = 20.5 to 320.9%) per year.

Notably, there were countries with contrary trends of malaria incidence or mortality rates according to the EAPCs estimated by the two models (from 2000 to 2019, and from 2015 to 2019). Malaria incidence rates were mostly negative from 2000 to 2019, except during the 2015 to 2019 period in Angola, Burundi, Chad, Comoros, Congo, Cote d'Ivoire, Democratic Republic of the Congo, Nigeria, Yemen, Costa Rica, and Ecuador. Trends were mostly positive from 2000 to 2019, except for downtrends from 2015 to 2019 in Niger. Malaria mortality rates were mostly negative from 2000 to 2019, except in the period from 2015 to 2019 in Comoros, Sudan, and Yemen (**Figure 5**).

### IRR, MRR, and predicted malaria cases and deaths in 2020

Globally, during the COVID-19 pandemic in 2020, the excess number of malaria cases was 18.0 million by Model A, with the largest expansion in Nigeria (6.5 million), followed by DR Congo (3.7 million) and Angola (3.1 million); and 7.4 million by Model B, with the largest expansion in Ethiopia (2.5 million), followed by Nigeria (2.2 million) and Madagascar (1.9 million) (**Figure 1**). There were 83 291 excess malaria deaths across the world in 2020 by model A, with the largest excess death number in Nigeria (29 832 excess deaths), followed by DR Congo (16 391 excess deaths) and Tanzania (5958 excess deaths). For model B, there were 33 528 excess malaria deaths across the world, with the largest excess death number in DR Congo (8653 excess deaths), followed by Mozambique (4101 excess deaths) and Uganda (2187 excess deaths) (**Figure 2**).

The reported malaria incidence rates in 2020 in most countries were higher than the predicted incidence rates by model A (60%, 49 out of 81 countries) and Model B (52%, 42 out of 81 countries) (**Figure 3**). The reported malaria mortality rates in 2020 in most countries were also higher than the predicted mortality rates by model A (68%, 45 out of 66 countries) and model B (76%, 50 out of 66 countries) (**Figure 4**). For model A, the highest IRR was 35.6 in Costa Rica, followed by 10.2 in Nicaragua and 8.8 in Ecuador, and the highest MRR was 580.0 in Ecuador, followed by 6.3 in Botswana and 4.4 in the Philippines. For model B, the highest IRR was 15.6 in Bhutan, followed by 4.6 in the Philippines and 4.2 in Honduras, and the highest MRR was 58.0 in Ecuador, followed by 9.9 in Botswana and 4.5 in the Philippines.

## DISCUSSION

To the best of our knowledge, this is the first comprehensive effort to assess the excess incidence and mortality of malaria in 2020 during the COVID-19 pandemic, using data from the WHO Global Health Observatory Data set. Our results showed that during the COVID-19 pandemic, there were millions of excess malaria cases and tens of thousands of excess malaria deaths all over the world in 2020. Malaria incidence rates increased excessively in over 50% of the malaria-endemic countries, and malaria mortality rates also increased excessively in around 70% of the malaria-endemic countries where deaths due to malaria had occurred. Additionally, the global malaria burden was still huge and there were countries with contrary trends of malaria incidence or mortality rates according to the EAPCs estimated by our two study models (downtrends between 2000 and 2019 and uptrends between 2015 and 2019), indicating that the progress had stalled or trends reverted for global malaria incidence and mortality. With the large burden and complex situation of global malaria under the COVID-19 pandemic, multifaceted and multisectoral actions are needed to reverse the wrong direction of malaria incidence and mortality trends in certain countries and to eliminate malaria globally.

We found that during the COVID-19 pandemic in most malaria-endemic countries, the reported malaria incidence/mortality rates were higher than the predicted rates. During the COVID-19 pandemic, both health systems and services and individual behaviours were greatly affected by the pandemic, resulting in continued service disruptions as multiple waves of the SARS-CoV-2 transmission put increasing strain on health systems and economies, especially in those countries with great COVID-19 burden [1,16]. Moreover, government policy responses to this pandemic, including the reorganization of health systems and restrictions on movement, had also critically influenced disease control and deaths [17,18]. In 2020, with support from global, regional, and local patterners, concerted efforts were taken by malaria-endemic countries to mitigate disruptions to malaria services during the COVID-19 pandemic [19-21], However, delivery of malaria services was still challenging. In the essential health service pulse survey conducted by WHO in September 2020, among the 48 malaria-endemic countries that responded to the survey, six countries reported severe disruptions (≥50%) and 15 had partial disruptions (5 – 50%) [1]. Medical settings which previously provided communicable disease-related services were diverted to provide COVID-19-related services, and health care workers were also redeployed to support COVID-19-related services [22,23]. It was vital to determine how to support countries to maintain essential malaria services during the COVID-19 pandemic.

Malaria prevention services were also disrupted. Most malaria episodes and deaths are reported widely in the African continent, but Sub-Saharan African countries fell into a cycle of poverty and disease due to the depletion of human capital and finance, thus being less able to finance or sustain intervention programs, leading to higher malaria prevalence [24]. The disruption to malaria prevention services such as insecticide-treated net (ITN), indoor residual spraying, and seasonal malaria chemoprevention (SMC) due to the COVID-19 pandemic even made the problem worse in African countries. Less than 25% of ITNs had been distributed by the end of 2020 in Eritrea (0%), Kenya (1%), and Zambia (25%), and only about 60% of targeted SMC treatment doses were delivered in Gambia [1]. It was imperative that the RTS, S Malaria vaccine should be rolled out for use, and all sectors of the economy needed to act to alleviate and eventually eradicate the malaria burden through vaccines in Africa [25].

Another worrisome finding of our study is that some countries had reversed trends of malaria incidence or mortality rates according to the EAPCs estimated by the two models of our study (downtrends between 2000 and 2019 and uptrends between 2015 and 2019). Although the burden of malaria in 2000 was huge and its decrease was predictable, the uptrends of malaria burden between 2015 and 2019 in certain countries were unexpected since the Global Technical Strategy for Malaria 2016-2030 (GTS) was proposed by WHO in 2015 [2]. A previous study showed that most countries with higher malaria incidence rates in 2019 than in 2015 were in Sub-Saharan Africa (Cabo Verde, South Africa, The Kingdom of eSwatini, etc.), South America (Colombia, Ecuador, Venezuela, etc.), and South Asia (Pakistan and Afghanistan) [4]. WHO introduced the “1-3-7” approach to malaria surveillance to be a guideline to instruct malaria control programs worldwide, especially in countries or regions where malaria is close to elimination [26]. This approach aimed to have all cases of malaria reported within one day, case investigations conducted within three days, and in-depth investigations conducted within seven days [27]. It was clear that countries should adapt the global strategy to their local context and maximize the implementation of known effective measures; however, this was difficult in countries with weak health systems. Therefore, financial and policy support for these countries is needed to establish surveillance systems that can accurately and reliably track the burden of malaria, the interventions to reduce it, and the impact achieved geographically and temporally [28].

Our study had several limitations. First, our findings were heavily dependent on the data source. Data reported by WHO was estimated by several models fitted to the local malaria prevalence could hugely differ from the real malaria burden [1]. Therefore, our prediction based on the estimated data might have high uncertainty. Nevertheless, our findings could alert the world that during the COVID-19 pandemic, not only was reducing the impact of COVID-19 on public health and health systems vital, but also ensuring quality health services for pre-existing diseases. Second, our analysis excluded three countries where reported malaria incidence rates were zero in 2019, so their potential effect on our results is unknown. Third, the health consequences of malaria largely differed through different periods [4], but we failed to assess the impact of the COVID-19 pandemic on malaria in different age groups due to data limitations.

## CONCLUSIONS

Since 2015, the progress had stalled or trends were reverted for malaria incidence and mortality at the global level. The emergence of the COVID-19 pandemic indirectly caused an increase in the prevalence of malaria and thwarted the progress in malaria control. In 2020, there were millions of excess malaria cases and tens of thousands of excess malaria deaths all over the world during the COVID-19 pandemic. Global efforts to control the impact of the COVID-19 pandemic should be balanced with malaria control to to accomplish the GTS global malaria elimination goal.

## Additional material


Online Supplementary Document
