# Supplementary material for: Millions of excess cases and thousands of excess deaths of malaria occurred globally in 2020 during the COVID-19 pandemic
Source: J Glob Health. 2022 Dec 17;12:05045. doi: 10.7189/jogh.12.05045 (PMC9757497; doi:10.7189/jogh.12.05045)
Supplement: Online Supplementary Document [file jogh-12-05045-s001.pdf]

ONLINE SUPPLEMENTARY DOCUMENT

Title: Millions of excess cases and thousands of excess deaths of malaria occurred globally in 2020 during the COVID-19 pandemic

Authors: Qiao Liu, Wenxin Yan, Chenyuan Qin, Min Du, Min Liu, Jue Liu

Location	Incidence Rate (per 1000 population) in 2020			Mortality Rate (per 100,000 population) in 2020		
	Reported by WHO (95% UI)	Model A (95% CI)	Model B (95% CI)	Reported by WHO (95% UI)	Model A (95% CI)	Model B (95% CI)
**Africa**						
Angola	251.6 (166.9, 363.9)	157.43 (130.62, 189.74)	244.36 (228.32, 261.51)	48.65 (30.41, 87.52)	31.43 (26.94, 36.67)	42.82 (38.36, 47.8)
Benin	388.3 (274.3, 535.1)	382.98 (353.77, 414.61)	414.3 (362.39, 473.64)	83.5 (69.49, 103.9)	85.29 (75, 96.99)	77.72 (70.17, 86.07)
Botswana	1.13 (0.79, 1.7)	0.3 (0.14, 0.65)	0.33 (0.01, 10.66)	0.26 (0, 0.58)	0.04 (0.01, 0.13)	0.03 (0, 3.67)
Burkina Faso	389.9 (241.2, 593.8)	378.44 (348.56, 410.88)	345.96 (306.24, 390.82)	95.58 (68.61, 139.3)	87.28 (78.25, 97.34)	87.68 (83.58, 91.97)
Burundi	294.9 (179.2, 457.7)	171.94 (128.15, 230.68)	344.02 (301.56, 392.47)	48.96 (39.79, 64.76)	34.12 (27.97, 41.63)	46.55 (34.9, 62.1)
Cameroon	260 (169.8, 381.3)	220.28 (201.81, 240.44)	239.06 (235.43, 242.75)	55.91 (46.43, 68.53)	43.69 (39.31, 48.56)	47.49 (42.77, 52.74)
Central African Republic	336 (172, 600.5)	320.85 (309.58, 332.53)	314.87 (304.1, 326.03)	105.2 (60.98, 193.8)	100.05 (87.31, 114.64)	85.82 (80.83, 91.11)
Chad	206.3 (115.1, 349.4)	186.45 (176.25, 197.25)	210.1 (199.73, 221)	76.42 (57.52, 102.1)	76 (72.24, 79.96)	72.41 (69.52, 75.42)
Comoros	5.23 (5.23, 5.23)	5.69 (2.09, 15.49)	49.88 (9.3, 267.42)	1.26 (0, 2.07)	1.32 (0.46, 3.81)	13.51 (1.7, 107.49)
Congo	213.2 (130.8, 331)	167.42 (149.2, 187.87)	231.8 (212.53, 252.82)	42.66 (34.89, 61.09)	32.96 (28.87, 37.63)	41.59 (39.09, 44.24)
Cote d'Ivoire	287 (154.9, 488.2)	244.26 (213.11, 279.97)	289.83 (280.62, 299.34)	60.33 (46.1, 84.8)	48.94 (40.99, 58.43)	59.81 (40.08, 89.25)
Democratic Republic of the Congo	324.2 (248.6, 415.1)	283.29 (263.45, 304.62)	331.19 (317.1, 345.92)	92.13 (59.54, 146)	63.8 (55.19, 73.75)	77.17 (65.87, 90.41)
Equatorial Guinea	240.8 (135.7, 394.2)	276.91 (258.57, 296.55)	229.07 (214.2, 244.98)	48.04 (37.14, 67.07)	45.83 (42.03, 49.96)	43.53 (40.09, 47.27)
Eritrea	44.8 (28.8, 62.37)	31.98 (19.1, 53.55)	61.56 (31.19, 121.5)	10.49 (0.37, 21.32)	5.94 (3.3, 10.7)	12.79 (5.54, 29.54)
Eswatini	0.72 (0.72, 0.72)	0.85 (0.39, 1.87)	1.25 (0.23, 6.71)	0.00 (0.00,0.00)	..	..
Ethiopia	54.13 (29.93, 81.04)	62.79 (44.16, 89.3)	21.79 (14.57, 32.59)	12.07 (0.48, 26.16)	12.19 (9.06, 16.41)	5.24 (4.28, 6.41)
Gabon	215.5 (116.5, 367.5)	200.87 (146.11, 276.15)	191.67 (174.88, 210.07)	19.05 (14.78, 27.05)	16.71 (13.55, 20.6)	16.69 (16.07, 17.34)
Gambia	87.27 (61.36, 124.7)	91.17 (65.62, 126.68)	33.18 (13.99, 78.68)	25.45 (23.38, 28.34)	21.55 (19.82, 23.43)	24.24 (23.68, 24.81)
Ghana	162.8 (111.6, 228.1)	207.61 (176.82, 243.77)	135.12 (119.92, 152.24)	38.89 (36.01, 42.77)	37.69 (34.64, 41.02)	36.71 (31.56, 42.71)
Guinea	319.5 (194.1, 496.6)	356.05 (325, 390.08)	320.02 (303.04, 337.95)	77.78 (59.08, 96.66)	71.63 (61.46, 83.49)	60.12 (54.55, 66.27)
Guinea-Bissau	88.92 (24.45, 240.6)	50.55 (36.26, 70.47)	53.78 (37.08, 78)	51.88 (39.08, 79.57)	33.59 (26.17, 43.13)	46.46 (44.02, 49.04)
Kenya	50.93 (29.08, 82.79)	41.61 (32.24, 53.71)	53.93 (53.15, 54.72)	23.52 (21.28, 27.46)	19.43 (17.44, 21.64)	23.1 (21.8, 24.47)
Liberia	358 (211.7, 565.7)	330.59 (302.87, 360.84)	389.79 (335.56, 452.79)	90.97 (60.17, 122.5)	55.18 (42.92, 70.95)	84.51 (58.66, 121.74)
Madagascar	133.5 (99.64, 170.8)	63.95 (44.38, 92.14)	64.73 (32.15, 130.32)	34.16 (1.07, 64.96)	15.81 (10.97, 22.78)	16 (7.95, 32.21)
Malawi	228.5 (123.1, 387)	206.3 (188.47, 225.81)	195.11 (184.52, 206.32)	37.45 (32.16, 45.49)	31.48 (28.26, 35.08)	35.74 (35.11, 36.39)
Mali	357.5 (253, 507.7)	382.88 (358.71, 408.67)	342.17 (287.59, 407.11)	95.38 (74.11, 124.8)	78.89 (69.52, 89.53)	85.91 (58.03, 127.18)
Mauritania	29.99 (11.8, 53.85)	27.78 (18.64, 41.39)	25.23 (12.97, 49.11)	36.24 (29.01, 51.62)	34.28 (33.64, 34.94)	34.9 (34.61, 35.19)
Mozambique	320.2 (230.6, 429.8)	299.18 (286.85, 312.05)	294.43 (287.19, 301.84)	76.04 (51.37, 122.8)	58.3 (53.4, 63.65)	56.67 (51.54, 62.32)
Namibia	10.04 (7.94, 12.3)	4.46 (1.48, 13.46)	8.3 (0.15, 466.99)	2.53 (0.05, 4.66)	1.1 (0.36, 3.42)	2.07 (0.03, 122.22)
Niger	324.1 (157.3, 605.9)	397.18 (354.6, 444.88)	309.74 (304.18, 315.41)	72.03 (44.6, 128.7)	80.1 (70.25, 91.32)	60.45 (53.19, 68.72)
Nigeria	313.8 (225.4, 423.7)	282.35 (265, 300.83)	303.09 (300, 306.21)	96.87 (73.39, 133.3)	77.73 (72.43, 83.43)	95.97 (89.98, 102.36)
Rwanda	230.5 (169, 296.7)	274.63 (137.91, 546.88)	510.13 (152.51, 1706.34)	23.52 (21.96, 26.63)	18.11 (15.38, 21.33)	23.39 (22.88, 23.92)
Sao Tome and Principe	8.82 (8.82, 8.82)	6.03 (3.01, 12.08)	14.2 (10.78, 18.69)	0.00 (0.00,0.00)	..	..
Senegal	49.93 (33.9, 67.73)	29.89 (20.46, 43.67)	44.02 (20.34, 95.27)	27.48 (25.5, 30.81)	21.9 (19.25, 24.92)	27.42 (26.07, 28.84)
Sierra Leone	328.2 (174.6, 557.9)	366.79 (347.92, 386.68)	338.61 (329.49, 347.98)	101 (77.65, 128.6)	105.03 (88.75, 124.3)	73.89 (64.82, 84.24)
South Africa	0.75 (0.75, 0.75)	0.92 (0.59, 1.46)	1.09 (0.05, 22.36)	0.64 (0.63, 0.65)	1.26 (0.67, 2.39)	1.55 (0.07, 33.56)
South Sudan	286.9 (156.5, 483.5)	259.47 (249.23, 270.14)	285.08 (272.25, 298.52)	66.39 (39, 121.2)	45.38 (37.88, 54.37)	66.74 (61.04, 72.98)
Togo	228.9 (155.6, 321.6)	259.65 (235.34, 286.46)	199.76 (182.93, 218.14)	43.35 (35.78, 53.14)	44.71 (38.83, 51.48)	37.25 (29.45, 47.13)
Uganda	283.8 (187.6, 446.3)	253.19 (235.37, 272.36)	270.67 (213.03, 343.91)	47.44 (36.12, 67.62)	30.81 (26.19, 36.24)	41.15 (27.82, 60.88)
United Republic of Tanzania	120.2 (75.25, 182.3)	93.19 (82.21, 105.64)	103.91 (99.33, 108.7)	43.48 (38.61, 53.91)	32.27 (28.06, 37.1)	40.24 (39.41, 41.09)
Zambia	186.9 (119.6, 280.6)	153.86 (126.29, 187.44)	160.38 (146.31, 175.8)	48.66 (42.55, 56.44)	36.62 (31.05, 43.2)	42.75 (40.84, 44.76)
Zimbabwe	98.51 (59.09, 140.9)	69.97 (55.25, 88.6)	59.49 (21.11, 167.66)	25.21 (0.68, 53.16)	17.9 (14.14, 22.68)	15.22 (5.39, 42.95)
**South-East Asia**						
Bangladesh	0.43 (0.36, 0.49)	1.28 (0.91, 1.81)	0.71 (0.21, 2.4)	0.085 (0, 0.15)	0.3 (0.21, 0.43)	0.15 (0.04, 0.56)
Bhutan	0.04 (0.04, 0.04)	0.00 (0.00, 0.01)	0.00 (0.00, 0.00)	0.00 (0.00,0.00)	..	..
Democratic People's Republic of Korea	0.18 (0.18, 0.18)	0.29 (0.14, 0.6)	0.17 (0.09, 0.3)	0.00 (0.00,0.00)	..	..
India	3.22 (2.12, 4.63)	6.16 (5, 7.59)	3.59 (2.43, 5.3)	0.57 (0.039, 1.12)	1.01 (0.79, 1.29)	0.46 (0.26, 0.79)
Indonesia	2.87 (2.62, 3.16)	3.65 (2.71, 4.92)	1.82 (1.08, 3.05)	0.53 (0.036, 0.86)	0.66 (0.49, 0.89)	0.34 (0.19, 0.61)
Myanmar	2.55 (2.06, 3.06)	4.73 (2.52, 8.9)	1.76 (1.17, 2.65)	0.24 (0.037, 0.41)	0.77 (0.38, 1.6)	0.22 (0.16, 0.31)
Nepal	0.03 (0.02, 0.04)	0.19 (0.11, 0.32)	0.06 (0.01, 0.44)	0.00 (0.00,0.00)	..	..
Thailand	0.23 (0.23, 0.23)	0.46 (0.27, 0.8)	0.19 (0.15, 0.25)	0.023 (0.022, 0.024)	0.08 (0.06, 0.09)	0.07 (0.04, 0.13)
**Eastern Mediterranean**						
Afghanistan	8.44 (6.48, 10.7)	10 (6.05, 16.54)	19.18 (5.07, 72.58)	0.37 (0.14, 0.64)	0.4 (0.23, 0.72)	0.8 (0.19, 3.42)
Djibouti	97.62 (97.62, 97.62)	22.69 (11.65, 44.19)	81.44 (41.23, 160.87)	17.01 (1.21, 26.59)	4.69 (2.47, 8.91)	13.05 (4.23, 40.29)
Pakistan	2.5 (1.97, 3.34)	4.67 (3.47, 6.29)	2.61 (2.16, 3.15)	0.21 (0.042, 0.37)	0.35 (0.25, 0.5)	0.21 (0.14, 0.31)
Saudi Arabia	0.03 (0.03, 0.03)	0.01 (0, 0.02)	0.01 (0, 0.15)	0.00 (0.00,0.00)	..	..
Somalia	52.2 (30.91, 78.47)	34.52 (25.12, 47.42)	48.39 (43.67, 53.62)	13.36 (0.36, 29.75)	8.83 (6.43, 12.14)	12.39 (11.18, 13.72)
Sudan	73.4 (36.51, 131.5)	39.13 (29.32, 52.23)	72.93 (60.4, 88.07)	17.18 (0.59, 41.61)	9.22 (6.99, 12.17)	17.12 (16.75, 17.51)
Yemen	40.57 (28.55, 58.1)	32.07 (25.81, 39.85)	54.17 (40.46, 72.53)	10.29 (0.33, 21.03)	8.19 (6.6, 10.16)	13.69 (10.26, 18.27)
**Western Pacific**						
Cambodia	5.85 (5.04, 6.86)	13.3 (9.98, 17.72)	15.03 (4.3, 52.5)	0.36 (0.085, 0.6)	1.51 (1.09, 2.08)	0.97 (0.24, 3.98)
Lao People's Democratic Republic	1.5 (1.08, 1.98)	6.26 (3.87, 10.12)	1.75 (0.62, 4.94)	0.18 (0, 0.37)	0.78 (0.46, 1.33)	0.19 (0.07, 0.51)
Papua New Guinea	164.3 (112.7, 221.1)	125.77 (107.09, 147.71)	150.44 (93.62, 241.73)	33.11 (1.83, 64.27)	26.14 (22.47, 30.4)	29.21 (18.19, 46.9)
Philippines	0.68 (0.37, 2.86)	0.18 (0.12, 0.26)	0.15 (0.08, 0.29)	0.15 (0.005, 0.74)	0.03 (0.02, 0.05)	0.03 (0.02, 0.07)
Republic of Korea	0.1 (0.1, 0.1)	0.09 (0.06, 0.13)	0.12 (0.08, 0.19)	0.00 (0.00,0.00)	..	..
Solomon Islands	167.7 (146, 198)	66.99 (40.05, 112.04)	266.68 (108.9, 653.02)	18.24 (2.5, 29.85)	8.48 (5.29, 13.61)	29.7 (13.72, 64.28)
Vanuatu	2.96 (2.26, 3.99)	3.98 (2.15, 7.36)	4.09 (0.32, 52.9)	0.00 (0.00,0.00)	..	..
Viet Nam	0.02 (0.02, 0.03)	0.05 (0.03, 0.06)	0.04 (0.01, 0.08)	0.003 (0, 0.006)	0.01 (0, 0.01)	0.01 (0, 0.01)
**Americas**						
Bolivia (Plurinational State of)	3.12 (2.43, 3.83)	1.21 (0.94, 1.56)	1.87 (0.68, 5.09)	0.11 (0.038, 0.21)	0.04 (0.03, 0.05)	0.06 (0.02, 0.18)
Brazil	3.87 (3.47, 4.19)	3.23 (2.51, 4.17)	5.09 (2.47, 10.48)	0.097 (0.096, 0.098)	0.07 (0.05, 0.08)	0.1 (0.05, 0.21)
Colombia	9.41 (7.17, 11.77)	5.78 (4.35, 7.66)	9.99 (5.37, 18.57)	0.044 (0.043, 0.045)	0.06 (0.04, 0.09)	0.03 (0, 0.2)
Costa Rica	0.05 (0.05, 0.05)	0 (0, 0.01)	0.21 (0.06, 0.78)	..	..	..
Dominican Republic	0.17 (0.15, 0.2)	0.12 (0.06, 0.21)	0.17 (0.03, 1.09)	0.033 (0.032, 0.034)	0.03 (0.02, 0.06)	0.03 (0, 0.47)
Ecuador	3.76 (3.76, 3.76)	0.43 (0.17, 1.1)	5 (2.72, 9.19)	0.58 (0.57, 0.59)	0 (0, 0)	0.01 (0.01, 0.01)
Guatemala	0.09 (0.08, 0.11)	0.17 (0.12, 0.23)	0.15 (0.11, 0.22)	0.00 (0.00,0.00)	..	..
Guyana	28.17 (23.86, 32.73)	31.08 (21.42, 45.1)	43.01 (28.01, 66.03)	3.56 (0.25, 5.98)	4.11 (2.66, 6.35)	5.06 (2.87, 8.92)
Haiti	3.74 (2.48, 5.04)	2.57 (1.85, 3.56)	1.37 (0.59, 3.19)	0.95 (0.02, 1.91)	0.65 (0.47, 0.9)	0.35 (0.15, 0.83)
Honduras	0.12 (0.1, 0.15)	0.14 (0.09, 0.22)	0.03 (0.01, 0.09)	0.00 (0.00,0.01)	..	..
Mexico	0.13 (0.13, 0.13)	0.13 (0.1, 0.18)	0.29 (0.17, 0.51)	0.00 (0.00,0.00)	..	..
Nicaragua	11.5 (9.51, 13.65)	1.13 (0.34, 3.68)	13.3 (3.48, 50.86)	0.00 (0.00,0.00)	..	..
Panama	0.55 (0.53, 0.59)	0.14 (0.08, 0.28)	0.39 (0.18, 0.84)	0.00 (0.00,0.00)	..	..
Peru	2.3 (1.67, 3.2)	3.89 (2.71, 5.61)	2.87 (1.97, 4.18)	0.18 (0.031, 0.34)	0.28 (0.17, 0.46)	0.22 (0.17, 0.28)
Suriname	1.7 (1.69, 1.71)	0.33 (0.17, 0.63)	0.48 (0.03, 7.24)	0.00 (0.00,0.00)	..	..
Venezuela (Bolivarian Republic of)	16.3 (14.47, 18.77)	23.18 (14.67, 36.64)	54.48 (21.83, 135.98)	1.43 (0.26, 2.31)	2.32 (1.57, 3.41)	3.86 (1.44, 10.3)
**Color Key:**	Predicted rates were higher than the reported rates			Predicted rates were lower than the reported rates		

Supplementary Figure. Reported and predicted (by Model A and B) malaria incidence and mortality rates in 2020 in malaria-endemic countries. CI, confident interval. UI, uncertainty interval.

The online supplementary document (OSD) has a free format.

Submit the OSD as PDF.

If you have complex accompanying data or information that does not translate well into the PDF format (eg, datasheets or tools), consider using registers for such data, such as OSF or Zotero.

In the manuscript text, provide the link to the registered data.

Figures in the Online Supplementary Document should be numbered in this way: S1, S2, S3, etc., and cited in the text. Example: (Table S1 in the **Online Supplementary Document**).
